# 2-(*N*-Phenyl­methane­sulfonamido)­ethyl 1*H*-pyrrole-2-carboxyl­ate

**DOI:** 10.1107/S160053681101261X

**Published:** 2011-04-13

**Authors:** Salman Tariq Khan, Peng Yu, Aisha Nelofar, Zaheer Ahmed, Suchada Chantrapromma

**Affiliations:** a Pharmaceutical Research Centre, PCSIR Labs Complex, Karachi 75280, Pakistan; b Department of Pharmaceutical Engineering, Biotechnology College, Tianjin University of Science & Technology (TUST), Tianjin 300457, People’s Republic of China; c Faculty of Sciences, Department of Home and Health Sciences, Allama Iqbal Open University, H-8, Islamabad, Pakistan; d Crystal Materials Research Unit, Department of Chemistry, Faculty of Science, Prince of Songkla University, Hat-Yai, Songkhla 90112, Thailand

## Abstract

In the title compound, C_14_H_16_N_2_O_4_S, the eth­oxy­carbonyl group is nearly planar, with an r.m.s. deviation of 0.0067 Å, and is almost coplanar with the pyrrole ring [dihedral angle = 5.81 (15)°], whereas it is inclined at a dihedral angle of 61.90 (13)° to the phenyl ring. The dihedral angle between the pyrrole and phenyl rings is 56.15 (13)°. In the crystal, centrosymmetrically related mol­ecules are linked into dimers by pairs of N—H⋯O hydrogen bonds, forming rings of *R*_2_^2^(10) graph-set motif. The dimers are further connected by weak inter­molecular C—H⋯O hydrogen bonds and C—H⋯π inter­actions, forming layers parallel to the *bc* plane.

## Related literature

For the pharmacological and biological activity of pyrrole-2-carboxyl­ate derivatives and sulfonamides, see: Brienne *et al.* (1987 ▶); Burnham *et al.* (1998 ▶); Fan *et al.* (2008 ▶); Fu *et al.* (2002 ▶); Gupton *et al.* (1999 ▶); Manzanaro *et al.* (2006 ▶); Mayer *et al.* (2009 ▶); Yoshikawa *et al.* (1993 ▶, 1998 ▶). For a related structure, see: Khan *et al.* (2010 ▶). For standard bond-length data, see: Allen *et al.* (1987 ▶).
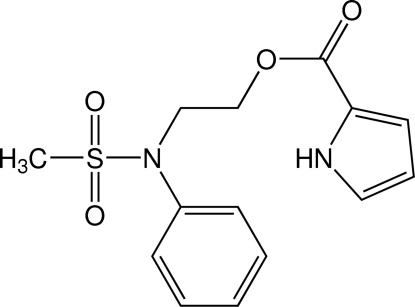


## Experimental

### 

#### Crystal data

C_14_H_16_N_2_O_4_S
*M* *_r_* = 308.36
Monoclinic, 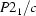

*a* = 12.186 (2) Å
*b* = 5.6516 (11) Å
*c* = 22.160 (4) Å
β = 104.47 (3)°
*V* = 1477.8 (5) Å^3^
*Z* = 4
Mo *K*α radiation
μ = 0.24 mm^−1^
*T* = 153 K
0.32 × 0.08 × 0.06 mm

#### Data collection

Rigaku Saturn CCD area-detector diffractometer
Absorption correction: multi-scan (*CrystalClear*; Rigaku, 2005 ▶) *T*_min_ = 0.928, *T*_max_ = 0.986
12044 measured reflections
3499 independent reflections
2726 reflections with *I* > 2σ(*I*)
*R*_int_ = 0.050

#### Refinement

*R*[*F* ^2^ > 2σ(*F* ^2^)] = 0.059
*wR*(*F* ^2^) = 0.152
*S* = 1.06
3499 reflections
196 parameters
H atoms treated by a mixture of independent and constrained refinement
Δρ_max_ = 0.43 e Å^−3^
Δρ_min_ = −0.50 e Å^−3^

### 

Data collection: *CrystalClear* (Rigaku, 2005 ▶); cell refinement: *CrystalClear*; data reduction: *CrystalClear*; program(s) used to solve structure: *SHELXTL* (Sheldrick, 2008 ▶); program(s) used to refine structure: *SHELXTL*; molecular graphics: *SHELXTL*; software used to prepare material for publication: *SHELXTL* and *PLATON* (Spek, 2009 ▶).

## Supplementary Material

Crystal structure: contains datablocks I, global. DOI: 10.1107/S160053681101261X/rz2576sup1.cif

Structure factors: contains datablocks I. DOI: 10.1107/S160053681101261X/rz2576Isup2.hkl

Additional supplementary materials:  crystallographic information; 3D view; checkCIF report

## Figures and Tables

**Table 1:** Hydrogen-bond geometry (Å, °) *Cg*1 is the centroid of the C2–C5/N1 ring.

*D*—H⋯*A*	*D*—H	H⋯*A*	*D*⋯*A*	*D*—H⋯*A*
N1—H1⋯O1^i^	0.92 (4)	1.99 (4)	2.894 (3)	167 (3)
C6—H6*B*⋯O4^ii^	0.99	2.53	3.415 (3)	148
C7—H7*A*⋯O4^iii^	0.99	2.55	3.406 (3)	145
C7—H7*B*⋯O1^iv^	0.99	2.54	3.431 (3)	150
C6—H6*A*⋯*Cg*1^v^	0.99	2.91	3.899 (3)	173

Symmetry codes: (i) 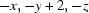; (ii) 
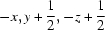
; (iii) 
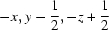
; (iv) 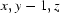; (v) 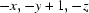.

**Fig. 1.**
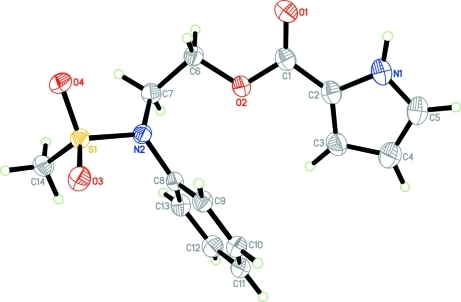
The molecular structure of the title compound, showing 50% probability displacement ellipsoids.

**Fig. 2.**
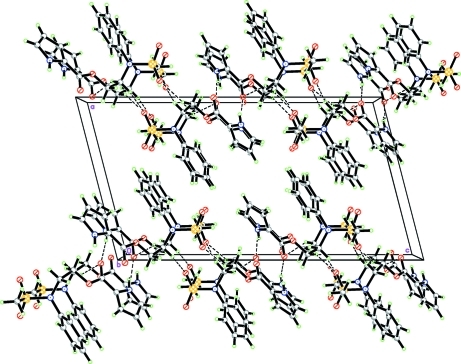
The crystal packing of the title compound viewd along the b axis. C—H···O weak interactions are drawn as dashed lines.

## supplementary crystallographic information

### Comment

Pyrrole-2-carboxylate based heterocyclic compounds, either naturally occurring or synthetic, have shown various pharmacological and biological activities such as anticancer (Burnham *et al.*, 1998; Gupton *et al.*, 1999; Fan *et al.*, 2008), antidiabetic, aldose reductase inhibition (Mayer *et al.*, 2009; Manzanaro *et al.*, 2006) anti-inflammatory and analgesic activities (Fu *et al.*, 2002). Likewise, compounds containing the sulfonamide moiety have their own biological importance as antifilarial (Brienne *et al.*, 1987) anti-inflammatory, antipyretic, analgesic and antiallergy agents (Yoshikawa *et al.*, 1993; Yoshikawa *et al.*, 1998) The title compound was synthesized as an intermediate which will be used in search of new potent anti-inflammatory and/or analgesic agents. Its crystal structure analysis was undertaken in order to establish the conformation of the various groups.

Fig. 1 shows the molecular structure of the title compound, in which the ethylcarboxylate unit (C1/C2/O1/O2/C6/C7) is planar with *r.m.s*. of 0.0067 (2) Å. This unit is almost co-planar with the pyrrole ring whereas is inclined to the benzene ring with dihedral angles of 5.81 (15) and 61.90 (13)°, respectively. The dihedral angle between the pyrrole and benzene rings is 56.15 (13)°. The orientation of the methylsulfonamide group (C14/S1/O3/O3/N2) with respect to the ethylcarboxylate unit can be indicated by the torsion angles S1–N2–C7–C6 = 121.87 (18)° and C14–S1–N2–C7 = 72.67 (18)°. The bond lengths are in normal ranges (Allen *et al.*, 1987) and comparable to those reported for a related structure (Khan *et al.*, 2010).

In the crystal structure (Fig. 2), N—H···O hydrogen interactions (Table 1) link centrosymmetrically related molecules into dimers forming rings of *R*^2^_2_(10) graph-set motif. The dimers are further arranged into layers parallel to the *bc* plane by weak intermolecular C—H···O hydrogen bonds and C—H···π interactions (Table 1).

### Experimental

The title compound was prepared by mixing 2-(phenylamino)ethyl-1*H*-pyrrole-2-carboxylate (1.0 g, 1.8 mmol), triethylamine (0.88 g, 8.8 mmol) and methanesulfonyl chloride (0.1 g, 8.8 mmol) in dichloromethane (6 ml) under nitrogen in sealed tube. The reaction mixture was stirred for 4 h at 273 K. The mixture was poured onto ice, and then sodium bicarbonate (10 ml, 10%) was added and the solution stirred for 10 minutes. The organic layer was separated and the aqueous layer was extracted with dichloromethane. The combined organic layers were dried over MgSO_4_, filtered and concentrated, yielding the a white precipitate of the title compound. Colourless needle-shaped single crystals of the title compound suitable for X-ray structure determination were recrystalized from dichloromethane by the slow evaporation of the solvent at room temperature after several days.

### Refinement

H atom attached to N1 was located in the difference Fourier map and refined isotropically. All other H atoms were placed in calculated positions with d(C—H) = 0.95 Å for aromatic, 0.99 for CH_2_ and 0.98 Å for CH_3_ atoms. The *U*_iso_ values were constrained to be 1.5*U*_eq_ of the carrier atom for methyl H atoms and 1.2*U*_eq_ for the remaining H atoms. A rotating group model was used for the methyl groups. The highest residual electron density peak is located at 1.13 Å from S1 and the deepest hole is located at 0.75 Å from S1.

### Crystal data

**Table:**

C_14_H_16_N_2_O_4_S	*F*(000) = 648
*M**_r_* = 308.36	*D*_x_ = 1.386 Mg m^−^^3^
Monoclinic, *P*2_1_/*c*	Mo *K*α radiation, λ = 0.71073 Å
Hall symbol: -P 2ybc	Cell parameters from 3499 reflections
*a* = 12.186 (2) Å	θ = 1.7–27.8°
*b* = 5.6516 (11) Å	µ = 0.24 mm^−^^1^
*c* = 22.160 (4) Å	*T* = 153 K
β = 104.47 (3)°	Needle, colourless
*V* = 1477.8 (5) Å^3^	0.32 × 0.08 × 0.06 mm
*Z* = 4	

### Data collection

**Table:**

Rigaku Saturn CCD area-detector diffractometer	3499 independent reflections
Radiation source: rotating anode	2726 reflections with *I* > 2σ(*I*)
multilayer	*R*_int_ = 0.050
Detector resolution: 14.63 pixels mm^-1^	θ_max_ = 27.8°, θ_min_ = 1.7°
ω and φ scans	*h* = −16→14
Absorption correction: multi-scan (*CrystalClear*; Rigaku, 2005)	*k* = −6→7
*T*_min_ = 0.928, *T*_max_ = 0.986	*l* = −28→29
12044 measured reflections	

### Refinement

**Table:**

Refinement on *F*^2^	Secondary atom site location: difference Fourier map
Least-squares matrix: full	Hydrogen site location: inferred from neighbouring sites
*R*[*F*^2^ > 2σ(*F*^2^)] = 0.059	H atoms treated by a mixture of independent and constrained refinement
*wR*(*F*^2^) = 0.152	*w* = 1/[σ^2^(*F*_o_^2^) + (0.0745*P*)^2^ + 0.5129*P*] where *P* = (*F*_o_^2^ + 2*F*_c_^2^)/3
*S* = 1.06	(Δ/σ)_max_ = 0.001
3499 reflections	Δρ_max_ = 0.43 e Å^−^^3^
196 parameters	Δρ_min_ = −0.50 e Å^−^^3^
0 restraints	Extinction correction: *SHELXTL* (Sheldrick, 2008), Fc^*^=kFc[1+0.001xFc^2^λ^3^/sin(2θ)]^-1/4^
Primary atom site location: structure-invariant direct methods	Extinction coefficient: 0.026 (3)

### Special details

**Table:**

Geometry. All e.s.d.'s (except the e.s.d. in the dihedral angle between two l.s. planes) are estimated using the full covariance matrix. The cell e.s.d.'s are taken into account individually in the estimation of e.s.d.'s in distances, angles and torsion angles; correlations between e.s.d.'s in cell parameters are only used when they are defined by crystal symmetry. An approximate (isotropic) treatment of cell e.s.d.'s is used for estimating e.s.d.'s involving l.s. planes.
Refinement. Refinement of *F*^2^ against ALL reflections. The weighted *R*-factor *wR* and goodness of fit *S* are based on *F*^2^, conventional *R*-factors *R* are based on *F*, with *F* set to zero for negative *F*^2^. The threshold expression of *F*^2^ > σ(*F*^2^) is used only for calculating *R*-factors(gt) *etc*. and is not relevant to the choice of reflections for refinement. *R*-factors based on *F*^2^ are statistically about twice as large as those based on *F*, and *R*- factors based on ALL data will be even larger.

### Fractional atomic coordinates and isotropic or equivalent isotropic displacement parameters (Å^2^)

**Table:**

	*x*	*y*	*z*	*U*_iso_*/*U*_eq_	
S1	0.19337 (5)	0.19937 (10)	0.28338 (2)	0.02406 (19)	
N1	0.1780 (2)	0.9013 (4)	−0.01667 (10)	0.0354 (5)	
N2	0.19052 (16)	0.2462 (3)	0.21007 (9)	0.0252 (4)	
O1	0.01281 (16)	0.7777 (3)	0.04865 (9)	0.0394 (5)	
O2	0.12012 (14)	0.4664 (3)	0.08930 (7)	0.0325 (4)	
O3	0.29861 (14)	0.2895 (3)	0.32044 (7)	0.0301 (4)	
O4	0.08904 (14)	0.2914 (3)	0.29278 (8)	0.0314 (4)	
C1	0.0969 (2)	0.6576 (4)	0.05242 (10)	0.0295 (5)	
C2	0.1832 (2)	0.7006 (4)	0.01917 (10)	0.0299 (5)	
C3	0.2777 (2)	0.5733 (5)	0.01512 (11)	0.0371 (6)	
H3	0.3024	0.4271	0.0351	0.045*	
C4	0.3301 (3)	0.7006 (5)	−0.02406 (13)	0.0449 (7)	
H4	0.3972	0.6562	−0.0356	0.054*	
C5	0.2671 (2)	0.9020 (5)	−0.04295 (12)	0.0423 (7)	
H5	0.2834	1.0208	−0.0698	0.051*	
C6	0.0391 (2)	0.4147 (4)	0.12552 (11)	0.0304 (5)	
H6A	−0.0368	0.3842	0.0977	0.036*	
H6B	0.0333	0.5495	0.1531	0.036*	
C7	0.08226 (19)	0.1984 (4)	0.16350 (11)	0.0280 (5)	
H7A	0.0249	0.1447	0.1852	0.034*	
H7B	0.0937	0.0696	0.1354	0.034*	
C8	0.29508 (19)	0.2205 (4)	0.19055 (10)	0.0231 (5)	
C9	0.37363 (19)	0.4032 (4)	0.20276 (10)	0.0263 (5)	
H9	0.3599	0.5392	0.2250	0.032*	
C10	0.4723 (2)	0.3868 (4)	0.18253 (11)	0.0308 (5)	
H10	0.5267	0.5107	0.1913	0.037*	
C11	0.4913 (2)	0.1904 (4)	0.14962 (11)	0.0329 (6)	
H11	0.5583	0.1806	0.1352	0.040*	
C12	0.4133 (2)	0.0074 (5)	0.13752 (11)	0.0341 (6)	
H12	0.4270	−0.1277	0.1150	0.041*	
C13	0.3151 (2)	0.0214 (4)	0.15835 (10)	0.0295 (5)	
H13	0.2619	−0.1048	0.1506	0.035*	
C14	0.1928 (2)	−0.1101 (4)	0.29289 (11)	0.0316 (5)	
H14A	0.2586	−0.1786	0.2814	0.047*	
H14B	0.1231	−0.1759	0.2660	0.047*	
H14C	0.1962	−0.1478	0.3365	0.047*	
H1	0.121 (3)	1.012 (6)	−0.0206 (16)	0.065 (10)*	

### Atomic displacement parameters (Å^2^)

**Table:**

	*U*^11^	*U*^22^	*U*^33^	*U*^12^	*U*^13^	*U*^23^
S1	0.0248 (3)	0.0244 (3)	0.0239 (3)	0.0005 (2)	0.0077 (2)	−0.0002 (2)
N1	0.0445 (14)	0.0324 (12)	0.0288 (10)	−0.0029 (10)	0.0081 (10)	0.0032 (9)
N2	0.0218 (10)	0.0319 (10)	0.0218 (9)	−0.0014 (8)	0.0049 (7)	0.0011 (8)
O1	0.0375 (11)	0.0365 (10)	0.0439 (11)	0.0065 (8)	0.0097 (8)	0.0104 (8)
O2	0.0333 (10)	0.0382 (10)	0.0274 (8)	0.0055 (7)	0.0099 (7)	0.0094 (7)
O3	0.0290 (9)	0.0368 (10)	0.0237 (8)	−0.0073 (7)	0.0049 (7)	−0.0051 (7)
O4	0.0298 (9)	0.0341 (10)	0.0338 (9)	0.0056 (7)	0.0146 (7)	0.0006 (7)
C1	0.0337 (13)	0.0299 (13)	0.0222 (11)	−0.0022 (10)	0.0016 (9)	−0.0010 (9)
C2	0.0358 (13)	0.0310 (13)	0.0208 (11)	−0.0030 (10)	0.0031 (9)	−0.0015 (9)
C3	0.0432 (15)	0.0391 (15)	0.0300 (12)	0.0058 (11)	0.0109 (11)	0.0060 (11)
C4	0.0486 (17)	0.0525 (18)	0.0385 (15)	0.0028 (13)	0.0202 (13)	0.0049 (13)
C5	0.0499 (17)	0.0450 (16)	0.0353 (14)	−0.0049 (13)	0.0168 (13)	0.0042 (12)
C6	0.0265 (12)	0.0386 (14)	0.0264 (11)	0.0009 (10)	0.0074 (9)	0.0034 (10)
C7	0.0253 (12)	0.0307 (13)	0.0266 (11)	−0.0040 (9)	0.0034 (9)	0.0004 (9)
C8	0.0249 (11)	0.0241 (11)	0.0204 (10)	0.0007 (8)	0.0057 (8)	0.0026 (8)
C9	0.0283 (12)	0.0226 (12)	0.0284 (11)	−0.0008 (9)	0.0079 (9)	0.0006 (9)
C10	0.0266 (12)	0.0324 (13)	0.0343 (12)	−0.0013 (9)	0.0093 (10)	0.0061 (10)
C11	0.0306 (13)	0.0407 (15)	0.0301 (12)	0.0070 (10)	0.0125 (10)	0.0082 (11)
C12	0.0395 (14)	0.0352 (14)	0.0295 (12)	0.0060 (11)	0.0124 (10)	−0.0014 (10)
C13	0.0346 (13)	0.0259 (12)	0.0282 (11)	−0.0022 (9)	0.0084 (10)	−0.0012 (9)
C14	0.0322 (13)	0.0274 (12)	0.0352 (13)	0.0017 (9)	0.0085 (10)	0.0053 (10)

### Geometric parameters (Å, °)

**Table:**

S1—O3	1.4325 (17)	C6—C7	1.503 (3)
S1—O4	1.4363 (17)	C6—H6A	0.9900
S1—N2	1.6376 (19)	C6—H6B	0.9900
S1—C14	1.762 (3)	C7—H7A	0.9900
N1—C5	1.354 (3)	C7—H7B	0.9900
N1—C2	1.377 (3)	C8—C13	1.386 (3)
N1—H1	0.92 (3)	C8—C9	1.388 (3)
N2—C8	1.452 (3)	C9—C10	1.387 (3)
N2—C7	1.483 (3)	C9—H9	0.9500
O1—C1	1.215 (3)	C10—C11	1.379 (4)
O2—C1	1.342 (3)	C10—H10	0.9500
O2—C6	1.450 (3)	C11—C12	1.384 (4)
C1—C2	1.447 (3)	C11—H11	0.9500
C2—C3	1.379 (3)	C12—C13	1.389 (3)
C3—C4	1.399 (4)	C12—H12	0.9500
C3—H3	0.9500	C13—H13	0.9500
C4—C5	1.378 (4)	C14—H14A	0.9800
C4—H4	0.9500	C14—H14B	0.9800
C5—H5	0.9500	C14—H14C	0.9800
			
O3—S1—O4	119.10 (11)	C7—C6—H6B	110.4
O3—S1—N2	107.77 (10)	H6A—C6—H6B	108.6
O4—S1—N2	106.58 (10)	N2—C7—C6	111.57 (19)
O3—S1—C14	108.31 (11)	N2—C7—H7A	109.3
O4—S1—C14	108.16 (11)	C6—C7—H7A	109.3
N2—S1—C14	106.23 (11)	N2—C7—H7B	109.3
C5—N1—C2	108.9 (2)	C6—C7—H7B	109.3
C5—N1—H1	129 (2)	H7A—C7—H7B	108.0
C2—N1—H1	122 (2)	C13—C8—C9	120.2 (2)
C8—N2—C7	117.97 (18)	C13—C8—N2	121.0 (2)
C8—N2—S1	118.48 (15)	C9—C8—N2	118.8 (2)
C7—N2—S1	117.10 (15)	C10—C9—C8	119.9 (2)
C1—O2—C6	115.53 (18)	C10—C9—H9	120.0
O1—C1—O2	122.5 (2)	C8—C9—H9	120.0
O1—C1—C2	125.4 (2)	C11—C10—C9	119.9 (2)
O2—C1—C2	112.1 (2)	C11—C10—H10	120.1
N1—C2—C3	108.1 (2)	C9—C10—H10	120.1
N1—C2—C1	119.8 (2)	C10—C11—C12	120.4 (2)
C3—C2—C1	132.1 (2)	C10—C11—H11	119.8
C2—C3—C4	106.8 (2)	C12—C11—H11	119.8
C2—C3—H3	126.6	C11—C12—C13	120.0 (2)
C4—C3—H3	126.6	C11—C12—H12	120.0
C5—C4—C3	107.9 (2)	C13—C12—H12	120.0
C5—C4—H4	126.0	C8—C13—C12	119.6 (2)
C3—C4—H4	126.0	C8—C13—H13	120.2
N1—C5—C4	108.2 (2)	C12—C13—H13	120.2
N1—C5—H5	125.9	S1—C14—H14A	109.5
C4—C5—H5	125.9	S1—C14—H14B	109.5
O2—C6—C7	106.41 (18)	H14A—C14—H14B	109.5
O2—C6—H6A	110.4	S1—C14—H14C	109.5
C7—C6—H6A	110.4	H14A—C14—H14C	109.5
O2—C6—H6B	110.4	H14B—C14—H14C	109.5
			
O3—S1—N2—C8	37.3 (2)	C3—C4—C5—N1	−0.1 (3)
O4—S1—N2—C8	166.21 (16)	C1—O2—C6—C7	−179.25 (19)
C14—S1—N2—C8	−78.62 (19)	C8—N2—C7—C6	−86.7 (2)
O3—S1—N2—C7	−171.42 (16)	S1—N2—C7—C6	121.87 (18)
O4—S1—N2—C7	−42.50 (19)	O2—C6—C7—N2	65.8 (2)
C14—S1—N2—C7	72.67 (18)	C7—N2—C8—C13	−47.9 (3)
C6—O2—C1—O1	−1.2 (3)	S1—N2—C8—C13	103.1 (2)
C6—O2—C1—C2	178.48 (19)	C7—N2—C8—C9	129.8 (2)
C5—N1—C2—C3	0.0 (3)	S1—N2—C8—C9	−79.1 (2)
C5—N1—C2—C1	−179.9 (2)	C13—C8—C9—C10	0.2 (3)
O1—C1—C2—N1	5.3 (4)	N2—C8—C9—C10	−177.6 (2)
O2—C1—C2—N1	−174.4 (2)	C8—C9—C10—C11	0.8 (3)
O1—C1—C2—C3	−174.7 (3)	C9—C10—C11—C12	−1.1 (4)
O2—C1—C2—C3	5.7 (4)	C10—C11—C12—C13	0.2 (4)
N1—C2—C3—C4	0.0 (3)	C9—C8—C13—C12	−1.0 (3)
C1—C2—C3—C4	179.9 (3)	N2—C8—C13—C12	176.7 (2)
C2—C3—C4—C5	0.1 (3)	C11—C12—C13—C8	0.8 (4)
C2—N1—C5—C4	0.1 (3)		

### Hydrogen-bond geometry (Å, °)

**Table:**

*Cg*1 is the centroid of the C2–C5/N1 ring.

**Table:**

*D*—H···*A*	*D*—H	H···*A*	*D*···*A*	*D*—H···*A*
N1—H1···O1^i^	0.92 (4)	1.99 (4)	2.894 (3)	167 (3)
C6—H6B···O4^ii^	0.99	2.53	3.415 (3)	148
C7—H7A···O4^iii^	0.99	2.55	3.406 (3)	145
C7—H7B···O1^iv^	0.99	2.54	3.431 (3)	150
C6—H6A···Cg1^v^	0.99	2.91	3.899 (3)	173

Symmetry codes: (i) −*x*, −*y*+2, −*z*; (ii) −*x*, *y*+1/2, −*z*+1/2; (iii) −*x*, *y*−1/2, −*z*+1/2; (iv) *x*, *y*−1, *z*; (v) −*x*, −*y*+1, −*z*.
